# The African Kelleni’s roadmap using nitazoxanide and broad-spectrum antimicrobials to abort returning to COVID-19 square one

**DOI:** 10.1007/s10787-023-01263-4

**Published:** 2023-06-16

**Authors:** Mina T. Kelleni

**Affiliations:** Pharmacology Department, College of Medicine, Minia University, El Minya, 61111 Egypt

**Keywords:** SARS CoV-2, Omicron variant, COVID-19, Nitazoxanide, Kelleni’s protocol, Broad-spectrum antimicrobials

## Abstract

For over 3.5 years, SARS CoV-2 is continuing to evolve threatening to return all and any improvement the world has made into square one. In this clinically oriented systematic review and perspective, the author explains how the best current medical evidence is strongly supporting the use of the low cost, widely available and very safe nitazoxanide in early management of COVID-19, debates the relevant theoretical studies that negated or doubted this benefit, and suggests an African roadmap to preempt the worst-case scenario if or when a new SARS CoV-2 (sub) variant or even a new respiratory virus causes a new global surge of morbidity and mortality. Kelleni’s protocol, including nitazoxanide as an integral component, is continuing to perfectly save lives of patients infected with many viruses, including SARS CoV-2 and the author stresses that respiratory RNA viruses are best managed with early pharmacological treatment. Broad-spectrum antimicrobials as nitazoxanide and azithromycin together with other therapeutics as non-steroidal anti-inflammatory drugs and the antihistaminic loratadine should be considered first to personalize the clinical management of COVID-19 and selected other alarming viral infections.

After 3.5 years, COVID-19 morbidity and mortality especially in the most vulnerable groups of patients have not stopped, though globally encountered in a significantly lower incidence than at the start of the pandemic. Nonetheless, many countries are still struggling to return back to near normal life as described before November 2019 and some notorious COVID-19 mandates are still adopted in some of them. However, most countries have finally moved forward wishing to start the much anticipated COVID-19 final countdown (Kelleni 2022a), unless for the risk coming from the continuous evolution and changing tropism of SARS CoV-2 omicron subvariants threatening to return the whole world into to square one (Kelleni 2023c). Therefore, it is of utmost importance to at least maintain the current COVID-19 status quo and to defend the global improvement regarding the quality of life that has occurred in the past few months, if the world is really determined to keep going forward. Therefore, the threat to return to COVID-19 square one is always lingering unless we decide to preempt the worst-case scenario.

Notably, I have recently called the global health care authorities for aborting the remaining COVID-19 restrictions/mandates (Kelleni 2023a, d) and emailed top officials in the US FDA and the WHO informing about this call. Fortunately, we soon witnessed a positive response represented by the subsequent official termination of the American and the World Health Organization COVID-19 public health emergency status in April and May 2023, respectively.

However, in our clinical practice, we continue to witness the ongoing evolutionary changing tropism of SARS CoV-2 omicron subvariants (Kelleni 2023c) including the recent XBB.1.16 subvariant, currently known with its evolved enhanced tropism toward the conjunctival epithelia especially in the pediatric population. Moreover, many international experts anticipate a new surge of cases coming from one of the currently evolving or a yet to be discovered SARS CoV-2 (sub)variants and some even warned that this could be soon witnessed, even sooner than what was previously expected (Kelleni 2023d).

Hence, I would like to suggest that an African roadmap adopting early pharmacological treatment using Kelleni’s protocol could be considered as the best antidote to abort any upcoming SARS CoV-2 variant(s) as it has showed not only theoretical (Kelleni 2020b, 2021b, c) but also remarkable real-world effectiveness (Kelleni 2021d, 2022a, 2023b, c) in the management of other respiratory viral infections.

Notably, for over 3 years, my parents who are in their eighth decade of life have enjoyed the freedom of living as prior to November 2019, and my two little daughters have likewise enjoyed learning and playing in their kindergarten, at least for most of the time except for 1 or 2 months of strict local governmental policies regarding mask mandates. My family and I trusted science and never abandoned good faith while scientifically choosing to adopt early treatment of the natural inevitable infection using Kelleni’s protocol, considering natural SARS CoV-2 infection as a safe, updated, and unpaid vaccine (Kelleni 2023a). Similarly, we trusted science (Kelleni 2021a) and chose to receive a single booster jab of BCG vaccine earlier in the pandemic (Kelleni 2022d). I suggest also that we were very scientific when we decided not to rush to receive the newly first ever approved for human mass vaccination mRNA or adeno-vectored SARS CoV-2 jabs (Kelleni 2021e) as other safer and more effective options, from our point of view, were and are available. Notably, since early in the pandemic, we have witnessed how a global consensus views might become rapidly shaped, then gradually proved wrong and we have also shared to show their fallacies (Kelleni 2020a).

Furthermore, I suggest that we, My family and I, kept our quality-of-life normal standard as prior to COVID-19 and throughout the pandemic, together with several hundreds of patients whom I directly managed their medical condition for over 3 years with 100% success rate even in the severe cases (Kelleni 2021d, 2022c), provided that they were dexamethasone/remdesivir naïve ones (Kelleni 2021f). Similarly, some Mexican physicians declared an astonishing 99% success rate when using nitazoxanide/azithromycin; two broad spectrum antimicrobials which were first suggested and adopted in Kelleni’s protocol, as they have also stated in their paper, to manage over 500 COVID-19 patients including over 300 patients suffering from severe condition (Romero-Cabello et al. 2023) and I argue that their adoption for prednisone as the third drug might have shared in their 1% loss (Kelleni 2021f). Unfortunately, millions of COVID-19 victims have lost their precious lives and billions of people were denied the same opportunity and right to keep living in a mandate-free world, at least until few months ago.

Notably, a randomized double-blind placebo-controlled clinical trial which enrolled 1092 participants and was performed in 36 centers in the U.S. showed that nitazoxanide has significantly reduced the risk of COVID-19 progression into severe illness by seven times, and it reduced the median time to sustained clinical recovery and time to return to usual health (Rossignol et al. 2022). Moreover, an earlier randomized, double-blind pilot clinical trial has also, using a five-point disease severity scale, proved the superiority of nitazoxanide over placebo while managing hospitalized COVID-19 patients suffering from mild respiratory insufficiency (Blum et al. 2021). Interestingly, when Patricia and colleagues showed a significant reduction in SARS CoV-2 viral load compared to placebo in a randomized, double-blind trial that assessed the role of nitazoxanide in the management of COVID-19, they should have followed the patients for a duration more than 5 days to elucidate the actual clinical outcome (Patricia et al. 2021) as has performed later when Rossignol and colleagues have followed the patients for 28 days (Rossignol et al. 2022) and as we have experienced in our clinical practice since April 2020 (Kelleni 2020b, 2021d). Notably, a recent randomized, placebo-controlled, single-blinded, parallel-group, pilot study has similarly demonstrated nitazoxanide anti-SARS CoV-2 activity with at least a 35% reduction in viral load at day 7 compared to placebo in mild–moderate COVID-19 patients and suggested outstanding potential clinical benefits (Silva et al. 2023).

Alarmingly, in some theoretical studies, we witness repeated obvious false scientific interpretations regarding the use of nitazoxanide to manage COVID-19, and we wish to stress the utmost importance of a concurrent clinical judgment especially during times of pandemics. For instance, though Qaseem and colleagues (Qaseem et al. 2022) have only referenced the previously discussed two studies (Patricia et al. 2021; Rossignol et al. 2022), they claimed that they reached a consensus that nitazoxanide should not be used to manage COVID-19 outpatients and we suggest that their conclusion is lacking any proper scientific justification as even shown by their cited references. Moreover, Vaz et al. (2023) have also only referenced two clinical trials previously discussed in this manuscript (Blum et al. 2021; Patricia et al. 2021), and yet, they doubted nitazoxanide effectiveness against COVID-19 and we would like to suggest that their judgment would be corrected when they consider other trials that were not cited or discussed and that their even their two cited references imply potential benefit that should have urged them to at least adopt a more cautious scientific impression. Similarly, though Weng et al.’s analysis has shown the superiority of nitazoxanide over placebo and standard of care regarding SARS CoV-2 eradication rate and acknowledged its excellent safety, they have unfortunately ignored the limitations of their study and recommended against its use in COVID-19 (Weng et al. 2022). Regrettably, even the authors who honestly admit some major limitations in their studies should have never concluded lack of evidence that supports the use of nitazoxanide to manage COVID-19, while even their cited references argue their conclusion (Martins-Filho et al. 2022), not to mention that other meta-analysis studies have shown proved clinical benefit as decreasing oxygen requirements and called for large-scale studies to elucidate other disputed benefits (Abuelazm et al. 2022). Importantly, we also highly urge all researchers to separate the trials using nitazoxanide for mild–moderate COVID-19 from those using it to manage severe COVID-19 (Rocco et al. 2022) in all future meta-analysis studies and to focus mainly on its early use to manage COVID-19. Interestingly, some excellent reviews have thoroughly discussed the potential and available clinical evidence when nitazoxanide is repurposed to manage COVID-19 (Al-kuraishy et al. 2022; Firth and Prathapan 2021; Kelleni 2022b; Lokhande and Devarajan 2021; Mahmoud et al. 2020; Prathapan 2022).

Thus, we would like to stress, from a clinical and academic point of view, that the low cost widely available generic nitazoxanide is more likely to be most clinically beneficial in aborting the ongoing progression of viral replication and it should be best considered, practiced, and (re)labeled as an early treatment broad spectrum antiviral drug (Kelleni 2023b, c). Nitazoxanide, together with azithromycin, NSAIDs and the antihistaminic loratadine (Kelleni 2023c), are my current trusted broad spectrum therapeutics that I use to personalize the clinical management of COVID-19 and other viral infections.

Finally, I would like to stress that it would be a huge unfortunate mistake if the same global authorities experienced panic when the anticipated new global wave of COVID-19 or other viral threat occurs. Meanwhile, it would be totally irresponsible if they choose to deliberately ignore that there was another scientific pathway, adopted by Africa, that could have spared the whole world the agony we all experienced, some while mourning the irreparable human losses and others while striving against the soaring inflation. We, humans, live in the same flying airship called earth and SARS CoV-2 remind us of our common vulnerability to both disease and tyranny, when we struggle against both, we simply do it because our own children and all beloved ones are on board, and we cannot afford to lose our one and only airship.

## Data Availability

My manuscript has no associated data.
